# Geography Matters, But… Evolving Success Factors for Nature-Oriented Health Tourism within Selected Alpine Destinations

**DOI:** 10.3390/ijerph18105389

**Published:** 2021-05-18

**Authors:** Jürgen Schmude, Markus Pillmayer, Maximilian Witting, Philipp Corradini

**Affiliations:** 1 Department of Geography, Ludwig Maximilian University Munich, Luisenstrasse 37, 80333 Munich, Germany; m.witting@lmu.de; 2 Department of Tourism, Munich University of Applied Sciences, Schachenmeierstrasse 35, 80636 Munich, Germany; markus.pillmayer@hm.edu; 3 Institute for Regional Development, Eurac Research, Viale Druso 1, 39100 Bolzano, Italy; philipp.corradini@eurac.edu

**Keywords:** health tourism, natural attraction, man-made attraction, evidence-based products, climate change, adaptation, alpine tourism, Alps

## Abstract

This paper analyzes the success factors of health tourism based on natural attractions in selected European spa and health destinations. The natural resources included in the offers, such as water, salt, and air, play a central role in this context, as their evidence-based effects have a high relevance for the health and wellbeing of tourists. Due to its specific geographical location and considering the threat of climate change, however, this offer is facing increasing challenges which make adaptation strategies necessary. In addition to a conceptional introduction to the topic, this paper contains a descriptive analysis of tourism statistics and the results from self-administered questionnaires with six selected representatives from alpine health destinations (DE, FR, IT, AT, CH, SI). The results show varying forms of health tourism based on natural attractions, which are also reflected in online marketing, with potential for optimization. The web research and the responses to the questionnaire revealed that evidence-based studies hardly play a role in promoting health touristic offers. Furthermore, climate change effects on natural attractions are considered extremely small and tend to prompt the development of new offers. Health destinations are advised to generate a clearer focus on the risks of climate change regarding natural resources.

## 1. Introduction

Recent years have seen a continuous increase in the significance and awareness of and regular preoccupation with personal health in business and society, particularly in Western industrial nations [1,2,3]. Demographic change towards an ageing population has a wide range of consequences, which exerts a strong influence on the demand for health-oriented products and services [4,5]. At the same time, health care reforms in recent decades and in close connection with the global wave of deregulation and liberalization have led to a drastic reduction in the range of services provided by social insurance funds [6,7]. This occurred alongside a broad decline in demand for many of Europe’s traditional spa and health resorts, which were largely unprepared for the structural transformations and continue to struggle with the consequences [8,9]. As a result, the corresponding health tourism destinations face the core challenge of adapting their products, which were previously utilized by customers who were mainly financed by social insurance funds, to make them equally attractive to self-payers. These self-paying guests are increasingly health tourists who, in addition to the desire to improve their health, have other motives for traveling—in particular, relaxation and the promotion of their general wellbeing. Their economic importance, or the importance of health tourism, can be seen, among other things, in the fact that, in 2014, around 5.8% of all the approximately 900 million domestic tourism trips in the EU-28 were health tourism trips. In terms of overnight stays, health tourism in the EU-28 comprises 233.7 million guest nights for domestic trips and 16.7 million international trips, totaling 250.4 million [2].

Recent decades have shown how man’s increasing alienation from his natural environment can have a diametric effect on health and wellbeing. With increasing urbanization, an increasing number of people are exposed to stress factors such as high noise and light emissions, traffic congestion, air pollution, increasing heat phenomena caused by an excessive density of buildings, pressure through interpersonal relationships and a fast-paced lifestyle; all these have negative effects on physiological and psychological health [10,11,12,13]. There is an increasing amount of evidence that exposure to and the associated direct contact with nature have many benefits: lifespan, general wellbeing, protection against cardiovascular disease, mental health, sleep duration or a generally quicker recuperation from illness are just a few aspects of this, which play an important role in this context and can be influenced positively [14,15,16]. Various authors [17,18,19], therefore, see an increasing significance of topics arising from the combination of natural resources, with scientifically proven added value for individual health.

Correspondingly, there is a paradigm shift in our understanding of health: rather than focusing exclusively on overcoming an illness by applying a selected medical therapy, it is now seen more in the light of prevention, with health-preserving or, ideally, health-promoting measures [9,20,21]. Taking a holistic view, the term health also encompasses aspects such as wellbeing, energy, dynamism and vitality [22,23,24]. In the course of this paradigm shift, the integrity and constant availability of natural resources is a conditio sine qua non for positioning within the international competition, especially in the segment of health tourism based on natural resources [21,25]. Here, there is a particular focus on those destinations which have a nature-based offer and are positioning themselves within the field of health tourism as part of the development of their destination [21,24,26]. Pertinent research projects emphasize the necessity of evidence-based health tourism based on natural resources. To date, however, there is a lack of comparative analyses focusing on the original offer of the destinations. The present paper aims to make a qualified contribution by bridging this gap.

Due to the different understanding of health tourism (see Section 2.1), this paper explores the role of evidence-based health tourism in six selected spa and health destinations in the alpine region. The natural resources must be the focus of evidence-based health tourism—thus, the question is how these natural resources change or need to change against the background of different geographical conditions and the influence of climate change. The paper also raises the question of how natural resources and the associated evidence-based health tourism are presented in the online marketing of the destinations. Therefore, the following research questions have been developed:

- How do tourism destinations perceive the application possibilities of evidence-based products?
- How do tourism destinations perceive the risks associated with climate change, relating to the natural resources utilized within the touristic offers?

The remaining part of this paper is structured as follows: Section 2 provides a profound literature review on the definition of health tourism, evidence-based offers and the challenges of climate change. Section 3 presents the data and the methods used in this study. The results are then presented in Section 4 and discussed regarding their meaning and relevance in Section 5. The paper concludes with a summary of the main findings, the study’s limitations, and an outlook for further necessary research in Section 6.

## 2. Literature Review

### 2.1. Definition of the Term Health Tourism

Health tourism was first defined by the International Union of Tourist Organizations (IUTO) [27], precursor to the United Nations World Tourism Organization (UNWTO), as “…the provision of health facilities utilizing the natural resources of the country, in particular mineral water and climate.” One of the most commonly used definitions of health tourism was provided by Kaspar [28], who described it as “…the sum of all the relationships and phenomena resulting from a change of location and residence by people in order to promote, stabilize and, as appropriate, restore physical, mental and social wellbeing while using health services and for whom the place where they are staying is neither their principal nor permanent place of residence or work.” While the above classification is useful when examining the demand side, it offers no insight into the development of health tourism products. Hall [29] specifically pinpoints this aspect and defines health tourism as “…a commercial phenomenon of industrial society which involves a person travelling overnight away from the normal home environment for the express benefit of maintaining or improving health, and the supply and promotion of facilities and destinations which seek to provide such benefits.”

Health tourism based on the application of natural resources is seen as a promising segment within a positive tourism and destination development [17,21,30,31]. Based on this, Steckenbauer et al. [20] foresee a clear upward trend towards the use of nature and natural remedies for health-oriented tourism. In this context, Schalber and Peters [32] emphasize that destinations with a rich supply of natural resources are particularly well-placed to benefit from this. The varying and sometimes conflicting interpretations of the health tourism center, in the broadest sense on the distinction between the terms “wellness”, meaning overall wellbeing in the context of health-promoting factors, and “medical”, meaning patients who, for various reasons, travel, sometimes across regional or national borders, to avail themselves of medical services outside their region or country of origin [3,19,33]. Above and beyond this, regional and national differences, interpretative approaches and schools of thought—depending on the scientific discipline—play an important role in the classification of health tourism. Against this multidisciplinary background, health tourism functions as an umbrella term for trips in which medical treatments and health services are a focus. The purpose of the stay is physical as well as psychological maintenance, stabilization and restoration of health. The spectrum of health-related and medical measures ranges from recreation, wellness and fitness to spa, rehabilitation and disease prevention to medical surgery, resulting in medical tourism, medical wellness, recreational tourism, spa tourism and wellness tourism, among other things [3,26,27,29,31,34].

### 2.2. Evidence-Based Health Tourism in the Alps

Health tourism in all its facets has become an independent tourism segment in the Alpine region in recent decades and has continuously gained in importance [32,35,36]. Due to the high demand for health tourism products, numerous destinations offer health tourism options. Generally, these offers make use of natural resources such as water or air, which are available locally [12,37,38]. Against this background, it is important to determine which natural resources actually offer the potential for health tourism and can be medically validated in clinical studies, since these resources, according to Niedermeier et al. [39], form the foundation of evidence-based health tourism and can be combined with hiking, walking or the outdoor experience in the broadest sense. Medical verifiability is essential in order to remove dubious or at least questionable offers within the spheres of health and wellness from the equation, which rather belong to the field of esotericism [40]. Patient-oriented decisions as well as the medical treatment of patients must be based on empirically proven efficacy. The potential offered by the combination of evidence-based products and health tourism tailored to these seems particularly considerable in the Alpine region, due to its natural characteristics [2,21,32,38].

Evidence-based proof of the effectiveness of health offers can generate competitive advantages in the medium- to long-term [20,21]. In this context, the decisive factor is not the health-oriented product alone, but the combination of the product with other offers available in the destination, which, in turn, represent unique selling points; these include, for example, cuisine, traditional practices or regional architecture [41].

### 2.3. The Challenge of Climate Change

In the future, these natural attractions, which are also referred to as the natural offer of a destination, will be exposed to the effects of climate change even more strongly than today. Overcoming these effects is a particular challenge for the various service providers and the associated development of the destinations [25,42,43,44,45]. At the same time, the effects of climate change pose a considerable threat to natural resources, such as water, salt, moor and air [12,37,38,46], particularly in view of the accumulation of extreme weather events (such as storms, fires and flooding) and prolonged periods of extreme climatic conditions (periods of drought, heatwaves lasting several months, etc.) [47,48]. The water quality, for example, can be massively impaired by an excessive concentration of various nutrient inputs (in particular nitrates), oils, herbicides etc. Similarly, ground-level pollution of the air with substances such as ozone, carbon monoxide, nitrogen oxides or aerosols can have adverse effects on the respiratory tract [49,50]. Various activities based on a natural attraction, for example, thermal mineral springs, low-allergen air or peat for mud baths, play a key role in the decision of whether or not to travel to a destination [51,52]. An impairment or threat to these resources through midge infestation, cyanobacteria or elevated pollutant levels can result in a negative perception from health tourists.

## 3. Materials and Methods

The study is based on six case studies, which are selected according to the following criteria: all destinations lie within the Alpine region, as defined by the Alpine Convention, and are referred to as spa resorts. The natural offer is also of relevance for tourism, that is, at least one health tourism product is bookable. In order to achieve the greatest possible geographical distribution, one case study was selected for Germany (DE), France (FR), Italy (IT), Austria (AT), Switzerland (CH) and Slovenia (SI), respectively (Section 3.1). 

**Figure 1:**
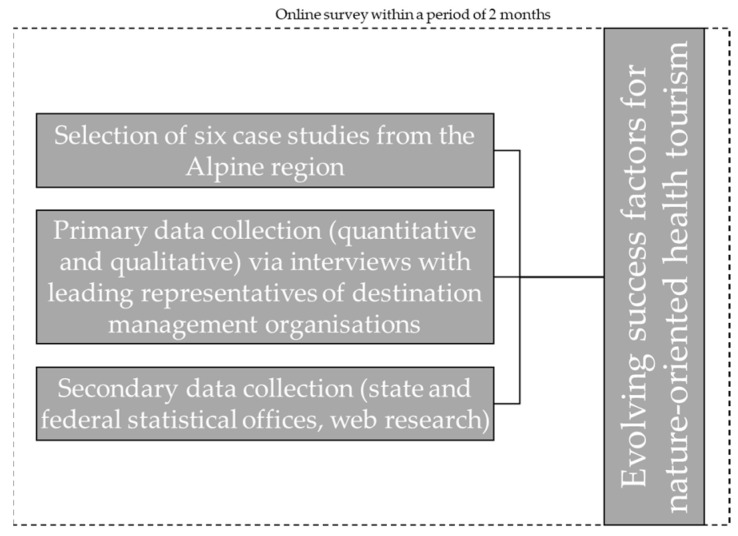
Methodological approach of the study.

From a methodological perspective, this paper consists of two parts (Figure 1 and Section 3.2). First, representatives of each selected destination were interviewed with regard to the current situation as well as the future development of health tourism and evidence-based health touristic offers in the destinations. In a second step, the information gathered through the interviews is supplemented by a descriptive analysis of secondary data (i.e., tourism statistics and web research) to create a profile of the selected case studies.

### 3.1. Research Area and Case Studies

**Figure 2:**
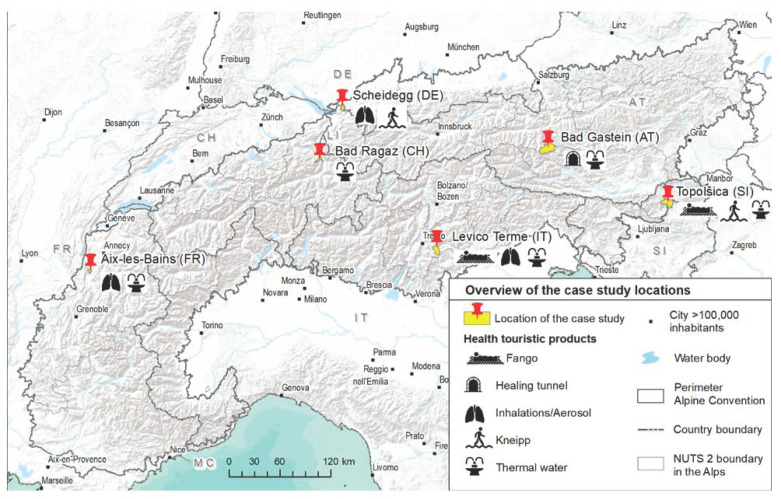
Selected case studies in the European Alps including natural offer and health touristic products. Source: Map based on [54,55,56,57,58].

The Alps, with the adjacent states France, Monaco, Italy, Switzerland, Liechtenstein, Germany, Austria and Slovenia, are a unique natural, cultural and economic area. They are characterized by a multitude of industries, including a declining alpine farming industry, and an expanding tourism economy [53]. This paper places a focus on selected destinations (Figure 2) in those countries which, based on their original offer, have a high potential for evidence-based health tourism, but currently only partially exploit or draw value from it. The following section and Table 1 describes the case studies examined in Germany, France, Italy, Austria, Switzerland and Slovenia.

Aix-les-Bains (France) lies in the region Auvergne-Rhône-Alpes in the Département of Savoie and had a total population of 30,272 in the year 2019. The health touristic offer is based on sulfur springs, whose water is used for drinking cures and hydrotherapy. The first spa facilities were established in the late 18th century. The number of overnight stays for Aix-les-Bains, only recorded uniformly since 2016 due to territorial reorganization, has remained constant between 1.9 million and 2.4 million (Table 2) [59,60].

The Austrian spa and winter sport resort Bad Gastein (3944 inhabitants in the year 2020) lies in the Hohe Tauern National Park in the federal state of Salzburg. The first evidence of the use of the thermal springs for medical purposes dates back to the 15th century. Since 1952, the health touristic offers, summarized under the term “Gasteiner Kur” (the Gastein cure), have been supplemented by the Gastein healing tunnel. In 2019, Bad Gastein recorded a total of approx. 1.12 million overnight stays, a high figure which has remained constant over the last 20 years [61,62].

The spa resort Bad Ragaz in the Swiss canton of St. Gallen (6494 inhabitants in the year 2020) is famous for its thermal waters, which have a temperature of 36.5 °C (97.7 °F). Discovered in the year 1240, the thermal waters represent the core of the health touristic offers in Bad Ragaz since the late 19th century. A comparison of the number of overnight stays in 2010 and 2019 reveals a decrease of approx. 25.5%, to 141,626 overnight stays (2019) [63,64].

The community of Levico Terme (Italy) lies in the region Trentino-South Tyrol and had 8133 inhabitants in 2019. The health touristic offer focuses primarily on the thermal baths, which came into being in 1860 with the founding of the “Società Balneare” (bathing society). In the year 2019, Levico Terme registered 1.05 million overnight stays—an increase of 12.7% compared to the year 2010 [65,66,67].

Located in the Allgäu region (Bavaria, Germany), and with 4302 inhabitants in 2019, Scheidegg has been a recognized mountain air health resort since 1936 and a Kneipp resort since 1973. Scheidegg is particularly famous for the cold-water therapy (Kneipp cures) developed by the priest and naturopath Sebastian Kneipp (1821–1897). In the year 2019, the resort registered a total of 550,412 overnight stays, an increase of about 21% since 2010 [68,69,70].

The health resort Topolšica (about 1300 inhabitants in 2020) lies in the northern Slovenian region of Savinja and is one of 15 natural spas in Slovenia. The health touristic offer includes the treatment of respiratory diseases and minor chronic cardiovascular diseases. Overnight stays have increased by approx. 6.9%, to 96,853 (2019), in comparison to 2015 (90,566) [71,72].

Table 1 shows the options for combining health touristic offers with other tourism offers in all case studies. Aix-les-Bains, but also Bad Ragaz and Bad Gastein, offer a very wide-ranging portfolio compared to Topolšica and Scheidegg. As the thermal baths in Levico are closed from November to April, no combination of health and winter tourism offers is possible there.

### 3.2. Survey Instrument and Data Collection

A self-administered survey provides the data basis of this study. The development of the quantitative questionnaire and the associated identification of relevant topics was carried out based on an in-depth review of the literature and an examination of the current state of research (Section 2). The questionnaire comprises 14 questions (11 semi-open, 3 closed) that refer to the health touristic products, providing an initial overview of the natural offer on-site. Closely connected to them are questions of possible combinations with other tourist activities such as hiking or cycling, creating a mixed offering and thus contributing to the attractiveness of the destination. (Table 1). A further topic area focuses on evidence-based studies to take the medical reliability of the natural offer into account (Section 2.2) for health tourism products. The demand side is also examined in detail based on the available statistics, which clearly underscore the importance of health tourism for the destinations. The questionnaire is completed by questions relating to climate change and the request for an assessment of the future development of evidence-based health tourism.

Using purposive sampling, the questionnaire was sent and completed by representatives of the Destination Management Organizations (DMOs) of the six selected destinations at the beginning of November 2020. These local experts have acknowledged expertise on the topics covered and the destinations examined. Furthermore, obtaining the opinion of local experts is always recommendable, as it provides exclusive insights into specific specialist knowledge, structural connections and processes on-site, which would otherwise remain inaccessible to outsiders [73]. Various touristic indicators (e.g., arrivals and overnight stays) and further information (e.g., the number of prescribed outpatient or inpatient treatments) were also requested.

In addition to the questionnaire, data from the official statistics of the countries in which the selected destinations are located were collected. It should be noted here that the national statistical concepts and their report groups vary. For this reason, not all data regarding the five case studies are available in an identical form. Where available with the desired degree of detail, data from the destinations were used and supplemented by data from a comparison with the official statistics from the countries. The tourism statistics used below refer to the years up to 2019, as the COVID-19 pandemic and the associated global downturn in arrivals and overnight stays render the data for the year 2020 unsuitable for the analysis of tourism development in the destinations. 

Finally, web research on marketing and the use of evidence-based studies for the promotion of health touristic offers on the websites of the destinations was conducted in January and February 2021. 

## 4. Results

The results of the study are presented in greater detail in the following section, in three content blocks: (a) comparative analysis of the touristic indicators and their development; (b) internet research regarding marketing and the use of evidence-based studies for the promotion of health touristic offers in the destinations; (c) destination-specific evaluation of the expected risks to the natural attractions and health tourism products resulting from climate change.

### 4.1. Comparative Analysis of Touristic Indicators

Select key touristic indicators are compared in Table 2. The number of total annual overnight stays in the three destinations in France, Austria and Italy is relatively high. In contrast, Scheidegg and, notably, Bad Ragaz and Topolšica, have a comparatively low number of annual overnight stays. In this context, however, it is important to take the respective size of the communities measured by the number of inhabitants into account—considering this, Bad Gastein, Levico Terme and Scheidegg have a particularly high tourism intensity, i.e., in these communities, tourism is of greater economic importance than in Bad Ragaz, Topolšica and Aix-les-Bains.

In addition to the marked differences in the annual number of arrivals and overnight stays, the variation in the guests’ average length of stay in the individual destinations is conspicuous. While guests spend a relatively short time in Aix-les-Bains and Bad Ragaz, they stay from two to five days longer in Levico Terme, Bad Gastein and Scheidegg. However, all destinations have one thing in common: the length of stay has decreased continuously over the last ten years, although this is a general trend within the tourism industry. Looking at the length of stay over the course of the year, a seasonal fluctuation can be seen in the destinations with a high length of stay: Bad Gastein (between 4.5 days in December and 10.6 days in November), Levico Terme (between 2.2 days in March or November and 7.6 days in July or August) and Scheidegg (between 6.2 days in May and 9.8 days in March). These fluctuations can primarily be explained by the varying demand for specific touristic products in different seasons.

Seasonality (=number of overnight stays in the month with the highest number of overnight stays/lowest number of overnight stays) is very high, particularly in Aix-les-Bains and Bad Gastein, but above all in Levico Terme. Analysis of the monthly share in the total annual overnight stays reveals that Aix-les-Bains and Levico Terme are summer destinations, whereas in Bad Gastein and Bad Ragaz, there are two peaks in the number of overnight stays (summer and winter). Placed in context, Bad Gastein has significantly more overnight stays in winter, whereas Bad Ragaz has slightly more overnight stays in summer. In Scheidegg and Topolšica, overnight stays are distributed relatively uniformly over all months, i.e., these two communities can be considered year-round destinations.

A comparison of the arrivals and overnight stays in the years 2019 and 2010 reveals different development trends. In Bad Ragaz, both overnight stays (−25.5%) and arrivals (−8.2%) decreased significantly, while figures remained relatively constant in Bad Gastein (−4.8% overnight stays; +7.5% arrivals). An appreciable increase can be seen in Levico Terme (+34.7% overnight stays; +12.7% arrivals) and Scheidegg (+113.1% overnight stays; +21.0% arrivals). Although the data basis for Aix-les-Bains (3.0% fewer overnight stays than in 2016) and Topolšica (6.9% more overnight stays than in 2015) is significantly more limited, these two destinations still exhibit a comprehensible trend regarding the overnight stays: stagnation in Aix-les-Bains and a continuous increase in Topolšica. The marked increase in the number of arrivals and overnight stays in Levico Terme, Scheidegg and Topolšica can be attributed to the shift in focus in health tourism—away from the classic spa stay and towards relaxation, wellness and wellbeing as the central motivations for travel. Further information can be seen in Table 2.

**Table 2:** Selected touristic indicators per case study 2019.

Destinations	Aix-les-Bains (FR)	Bad Gastein (AT)	Bad Ragaz (CH)	Levico Terme (IT)	Scheidegg (DE)	Topolšica (SI)
Overnight stays	1,923,430	1,118,205	141,626	1,045,947	550,412	96,853
Arrivals	976,360	196,320	54,634	222,719	72,698	N/A *
Length of Stay	2.0	5.7	2.6	4.7	7.6	N/A *
Tourism intensity	63,538	283,521	21,809	128,605	127,943	81,321
Seasonality	6.4 (Aug/Jan)	7.0 (Feb/Nov)	2.0 (Jul/Feb)	19.6 (Aug/Nov)	1.7 (Aug/Nov)	2.1 (Aug/Apr)
Monthly share of overnight stays	53% (Jun–Sep); 2.5% (Jan)	41% (Jan–Mar); 29% (Jun–Aug)	41% (Jul–Oct); 5.4% (Feb)	49% (Jul–Aug); 8.4% (Nov–Feb)	22% (Jul–Aug); 6 to 9% (rest)	14% (Aug); 6 to 9% (rest)
Type of destination	summer	summer/winter	summer/winter	summer	year-round	year-round
Significance of day tourism	low	low	high	high	low	low
Health touristic share	N/A *	35%	60%	6–8%	65%	12%

* not available. Source: own compilation based on [60,62,64,65,66,69,70,71].

On the one hand, an analysis of the age structure of guests in the destinations (Table 3) reveals the main target groups and, on the other, it allows for an identification of the regional source markets. Day tourists are of particular importance in Bad Ragaz and Levico Terme. The ratio of health tourists to total tourists provides information on the diversification of the tourism offers in the destinations. For example, Scheidegg und Bad Ragaz focus more strongly on health tourism than Aix-les-Bains, Levico Terme and Topolšica. For Bad Gastein, health tourism is a further important pillar in its product portfolio, supplementing the strong winter and summer tourism.

The analysis of the health tourists’ age structure is significant against the background of demographic change and shows that the destinations fall into two groups. In Aix-les-Bains, Bad Gastein, Bad Ragaz, Levico Terme and Topolšica, 55% to 80% of the guests are in an older age group (>50 years). Only Scheidegg has the age group 30–49 years as its main target group. The clear focus on the age group >50 years can be explained by its potential to generate demand, which is increasingly a result of demographic change [74]. Moreover, health and wellbeing are high priorities for persons in this age group [8], which also includes segments, with a high amount of available time (due to retirement) and income.

There are also clear differences in the sources’ markets, and thus the origin of the health tourists (Table 4): Aix-les-Bains, Bad Gastein, Levico Terme and Scheidegg are visited almost exclusively by domestic health tourists. In contrast, a not inconsiderable proportion of guests in Topolšica (30%) and, above all, in Bad Ragaz (40%) are foreign health tourists, with Bad Ragaz alone showing a high percentage of guests from countries at some distance from the Alpine region (22%). This may be an indication of an initial internationalization of guests which, due to their history, has already progressed further in the well-known, traditional spa and health destinations—which include Bad Ragaz and Davos—than in, for example, Scheidegg. Table 4 illustrates the importance of these differences for the acquisition of guests in health tourism.

### 4.2. Web Research on Marketing and Evidence-Based Studies

As digital channels have now become one of the most important sources of information, a web research was carried out to examine the marketing of the respective destinations with regard to the presence of health tourism elements and their positioning within the website structure. While Aix-les-Bains, Bad Gastein, Levico Terme and Scheidegg have their own destination websites, the sites of Bad Ragaz and Topolšica are subsites, embedded in the websites of larger destination structures [59,61,63,67,68,72]. Moreover, research showed that destination marketing through health tourism information varies greatly from destination to destination. In some cases, the positioning of health tourism keywords within the various levels of the sites (comprising the homepage and subpages) sometimes requires substantial navigation activities of the potential guest in order to identify health touristic offers. On this basis, the prominence of health tourism aspects (meaning the ease with which they are found) was classified as “low”, “medium” or “high” (Table 5). 

The destinations Bad Ragaz, Levico Terme and Scheidegg are primarily marketed based on their health tourism aspects on their respective homepages. Scheidegg is also attempting to address an additional, specific niche segment and create a unique selling point with the slogan “Gluten-free vacations in Scheidegg”. Accordingly, its prominence within the marketing was ranked as “high”. Despite the presence of the terms, “baths” and “thermal springs” on the homepages of Bad Gastein and Toplšica, their prominence was classified as “medium”, since the terms were not main aspects of the websites, but merely featured in a listing of the general tourist activities offered at the destination. Aix-les-Bains does no prominent marketing on the homepage and only draws attention to health tourism products on the second subpage level. This can be explained, for example, by the diversification of the offer and a focus on other market segments.

The destinations also vary in their use of evidence-based studies. The experts polled in Bad Gastein, Bad Ragaz and Levico Terme pointed out that the destination collaborates closely with scientific institutes. In Aix-les-Bains, Scheidegg and Topolšica, on the other hand, there were no studies, or none that the experts were aware of. This is also evident in the marketing of the destination, as only Bad Gastein, on the third subpage level, refers specifically to evidence-based studies, several of which can be accessed. There is no reference to evidence-based studies on the websites of the other destinations. According to the experts, none of the destinations plan to include such references in the future, for various reasons. Bad Gastein and Bad Ragaz see this as the responsibility of other parties. Levico Terme is currently waiting on a change within the legal conditions for the financing of health tourism, which will be introduced in 2021, although additional evidence-based studies are not a priority at present. Topolšica sees no necessity for these, as guests are sent to the destination exclusively by health insurance companies.

### 4.3. Evaluation of Climate Change Effects on Natural Resources and Touristic Products

The natural attractions and their use to generate touristic value are also always subject to the influence of external factors. This is true at present and will apply even more in future to the consequences of climate change, as the natural resources required for the health touristic offer in the destinations (e.g., water, air) are strongly affected by climate change. In the evaluation of risk, all destinations present a uniform picture: the effects of climate change on the natural offer of thermal waters are not viewed as a threat by the DMO experts. Without exception, the health tourism products are also seen as not being at risk due to climate change. Indeed, in Bad Gastein and Levico Terme, climate change is perceived as an opportunity. Bad Gastein expects the hot summers of the future to result in a greater influx of tourists, represented by the inhabitants of major towns seeking cooler air in the mountains [75]. The summer destination Levico Terme sees the general rise in temperature as an opportunity to create new offers for the mid-season.

## 5. Discussion 

Essentially, all the destinations examined have the same aim: successful, future-oriented development of the destination with a specific focus on health-oriented offers and health tourism. With the help of a clear positioning, in particular within the context of evidence-based health tourism, they can become active both on a national and international level and bring themselves to the attention of a health-conscious and health-oriented clientele. In order to reach this goal, DMOs are advised to collect data and analyze key tourism indicators as well as construct target-group-specific marketing. Furthermore, they need to take climate change and the associated consequences for the natural attractions into consideration.

### 5.1. Comparative Analysis of Touristic Indicators

A comparison of the average length of stay reveals that the length of stay in Aix-les-Bains, on average, two days, is extremely low. In view of this, the effectiveness of the health touristic offers for the guest must be critically examined. A longer length of stay would be recommendable, both from the standpoint of the service providers and the guests, in particular with regard to the effectiveness of the natural offer. A completely different situation can be seen in Scheidegg, for example, where the length of stay is comparatively high (over seven days), a fact which can be interpreted as positive. The remaining destinations lie between these two poles, while it was not possible to calculate a value for Topolšica due to the lack of arrival figures. These differences can partly be explained by the considerable difference between the length of stay for spa guests (15 and more nights) and wellness tourists (4–9 days) [33]. Furthermore, the general decrease in the length of stay throughout the tourism industry is also visible in the selected health destinations. Here, this can be partially explained by the increase in self-financed stays compared to stays financed by the social insurance carriers, the latter gradually decreasing in number [9]. Increasing the average length of stay is both desirable and recommendable in order to achieve added value in terms of an increase in health and wellbeing [76]. The protagonists in the destinations need to create tailor-made products—based on the natural attractions and with a variety of activities—which encourage the guest to stay longer. The spa town of Merano in South Tyrol, for example, serves as a benchmark, having achieved clear positioning in the wellbeing segment through diversification of its health touristic offers. The increasing number of requests for a know-how transfer from other European spa and health tourism destinations reflects this [30,77].

An increase in the average length of stay is also a key aspect in the context of current debates on overtourism and overcrowding, which have been further intensified by the COVID-19 pandemic [78,79]. Wherever possible, guests should not only be encouraged to go on day trips to the destination, but to stay there overnight. Tourism intensity, the number of overnight stays generated per 1000 inhabitants, is a closely related indicator here (see Table 2). The tourism intensity in Bad Ragaz, for example, is only around 21,000, whereas in Bad Gastein, it is over 280,000. The critical question of whether tourism intensity in Bad Gastein is already too high, which can manifest itself in the form of increased traffic volume, noise emissions or general environmental impact in the location, must be raised here. In the medium to long term, this could impair or, in the worst-case scenario, even damage the natural attractions. This could be further exacerbated by the effects of climate change, leading to decreasing demand and a decline in the number of health tourists, which would require immediate corrective actions. Rather than relying on quantitative growth in the form of a simple increase in arrivals and overnight stays, a qualitative approach could both extend the average length of stay and address new target groups. Accessing new source markets and new age segments can help to reduce the seasonality of the respective tourism destination, and thus distribute the flow of tourists more evenly across the year [77,78].

In turn, seasonality offers an insight into the nature of the destination. For example, with a value of roughly 20, Levico Terme (Table 2) reveals itself as a destination with offers that are only available for a very brief period, because the thermal baths are closed from November to April. This raises the question of whether it is possible to gear offers toward health-oriented guests, available over a longer period of time. Health is a commodity that should be considered throughout the year and not merely for a few months. There is also a year-round demand for health products [9,31]. The situation is very different in Scheidegg, for example, which has a seasonality of under 2.0, and can thus be classified as an all-season destination like Topolšica. This can be interpreted as a competitive advantage, as offers in the health segment are available more or less throughout the year and can be combined with a broad range of other activities such as hiking or cycling. This increases flexibility, particularly in the design of products, across the seasons. Moreover, it considerably improves the use of capacities both for the health care providers and accommodation establishments, making it possible to ensure a certain (economic) continuity within the specific tourism destination.

A further precondition for a successful, future-oriented development of health tourism is the ongoing recording and analysis of (health) tourism structures in the form of monitoring [80,81]. No information was provided for the health resort Aix-les-Bains, as no data were available. For one of the most important health resorts in France, it should be the state-of-the-art to document the significance of health tourism. In this context, the self-perception of Levico Terme is interesting. The resort reports that health tourism accounts for less than 10% of overall tourism in the destination, an extremely low value. However, Levico Terme sees itself as a health destination and positions itself as such. Scheidegg reports a relatively high share of 65% in overall tourism—followed by Bad Ragaz, with 60%. The alignment of the year-round destination Scheidegg, offering health options in combination with other activities, underscores this importance. It is worth mentioning at this point that, with its focus on gluten-free nutrition, Scheidegg has identified a niche for itself which could be exploited in collaboration with an institute supplying evidence-based studies. In this way, the destination could generate possible competitive advantages and distinguish itself from the rest of the field. However, in this context it needs to be considered that health tourism and the legislation on health tourism are different in each country.

### 5.2. Web Research on Marketing and Evidence-Based Studies

Despite the focus on health tourism and health offers, the topic only plays a limited role in the respective internet presence of the various destinations. Although health touristic keywords such as “health”, “thermal baths” or “thermal springs” are found on the homepages, only half the destinations granted a “high” prominence to the topic on their destination website. There is clear scope for development here, as, in light of its evidence-based relevance, the topic of health and wellbeing will continue to play a major role in the everyday life of existing and future guests, as there is an increasing focus on a healthy lifestyle, preventive healthcare and an ageing population—the process of transformation from the 1st to the 2nd health market will be consolidated [3,9].

With regard to their health touristic offers, the case studies are in various stages of the transformation process, but there does seem to be a general desire for modification or adjustment to changes in the legal framework conditions and in demand patterns. However, evidence-based studies, which could be an important element in the successful marketing of the health offers, have not often been used to date. The website of Bad Gastein mentions such studies on the third subpage level, while that of Topolšica contains only one reference. A clear commitment to health tourism would be expected and indeed desirable here, particularly in the areas of spa, health and wellbeing. However, it would then be necessary to take appropriate measures. This seems expedient, both in order to promote and market the natural offer and to protect the sensitive natural attractions, particularly in view of climate change.

### 5.3. Evaluation of Climate Change Effects on Natural Resources and Touristic Products

The results relating to climate change and the associated effects on the natural offer are sobering: the experts polled are of the opinion that the natural offer is and will remain largely unaffected by climate change. This must be considered a misconception. Climate change will affect the natural resources, particularly in the Alpine region—in differing levels of intensity and to a differing extent, depending on their geographical location [50,52,82,83]. Climate-change-induced heat waves can negatively affect thermal water resources, which means that destinations may not fulfil the guidelines for the respective predicate in the future [84]. This concerns all case studies under investigation since they base part of their health touristic products on thermal water. Furthermore, the appearance of pollen and a climate-change-induced deterioration in air quality are risks for climatic health resorts, such as Aix-les-Bains, Bad Ragaz and, Scheidegg [85]. The destinations will then face the question of whether it is, in fact, still possible to use the natural resources for evidence-based health tourism products. If the natural attractions become less effective, this will have far-reaching consequences for the health offers and health tourism overall in the respective destinations. Their attractiveness as spa or health destinations will presumably decrease. For those catering to tourists, it must be a priority to protect and maintain the natural resources in the face of climate change. In this context, further studies examining the influence of climate change on the natural attractions and the possible associated consequences are necessary.

## 6. Conclusions

Health tourism has been a major trend for some years, particularly in view of increasing health-awareness and the prevention of illness [8,14,86]. This trend is flanked by the process of transformation from the 1st to the 2nd health market, which increasingly forces health-conscious guests to foot the bill for health-related services themselves [9,87,88]. Spa and health destinations need to confront these changing market conditions and define appropriate responses. A central element here is the natural offer based on resources such as water, air, salt and peat, with evidence-based positive beneficial effects. However, these natural offers are at risk due to climate change [48,49,82,89]. Against this background, this study polled six experts from selected spa and health destinations in the region of the Alpine Convention, using a questionnaire. The first section of the questionnaire focused on the health tourism products, providing an overview of the natural attractions in the respective destinations. A further focus was on evidence-based studies, in order to take the medical reliability of the natural attractions for health tourism products into consideration. The questionnaire was rounded off by questions on climate change, and the respondents were asked to provide an estimation of the future development of evidence-based health tourism. This information was supplemented by data from the national statistics of the countries in question in order to shed more light on the demand side, using selected touristic indicators and internet research on marketing to provide a closer evaluation of the marketing of evidence-based studies.

The results have shown that the six spa and health destinations examined are extremely heterogeneous destinations, where health tourism plays a role with varying intensity. The further development of health tourism is a common goal of all destinations. There is no patent remedy; rather, the destinations must continue with the initiated process of transformation and define individual measures based on the original and derived offer. This can involve positioning the destination based on its competence for healthcare and the prevention of illness through the addition of new health touristic offers (e.g., forest bathing, yoga and anti-stress courses) [10,12,18,39,90]. The combination of health touristic offers with other tourist activities can support this process. Such diversification should be accompanied by corresponding marketing, which currently represents a deficit in the area of online marketing in all destinations. Diversified marketing placing a greater focus on evidence-based studies and stronger promotion of the topics of health and wellbeing could be helpful here. Additionally, these topics are gaining in significance during the current transformation from the 1st to the 2nd health market. 

In reference to the title of this study, “Geography matters, but”, the results also show that the geographical circumstances (including topography and natural attractions) are an important factor for the success of health tourism in the destinations. However, marketing should emphasize them more strongly, and they should be combined on the website with a language offer, adapted to the surrounding language area. Regarding climate change, the destinations need to rethink their approaches [43,47,49,51,89]. There must be a clearer focus on the associated challenges and the risk to the natural attractions in future. Only by doing this will the spa and health destinations be able to successfully participate in this segment in the medium and long term.

This study shows that case studies have the potential to expand and refine theory, especially when enriched with empirical evidence. For example, despite the obvious effects of climate change, interviewees believe that it will have virtually no impact on the natural resources, and thus on evidence-based health tourism. The various natural resources, which have different characteristics due to the geographical location, and a partly different understanding of what health tourism entails, contribute to this fact. However, this is already a weakness of the study and of case studies per se. The selection of just six destinations cannot be considered representative, though it does offer initial insight into a complex and highly relevant topic. 

For future surveys, it would be advisable to elicit the participation of at least one spa or health destination from each country in the Alpine Convention—and ideally, all spa and health destinations. A corresponding quantitative survey should also take all countries in the Alpine region into consideration. It would also be advisable to include relevant service providers of the destinations within the survey, for example, thermal baths, hotels, medical practices, environmental protection and nature conservation organizations in addition to the experts from DMOs and, ideally, all health service providers participating in the tourism value chain. Moreover, the guests’ perspective would be extremely relevant—after all, it is they who take advantage of the evidence-based offers in order to improve their health or wellbeing. In view of the complexity behind the non-uniform understanding of health tourism, the segment of health tourists must also be differentiated here. The present study only covers the demand side indirectly via various touristic indicators. The interrelationship between average length of stay and the beneficial effect of the natural attractions on health also presents an interesting subject of research. The effects of climate change on the natural attractions could be examined in greater detail in a broad-based study to better understand how climate change impacts health tourism. Individual, subjective perception of climate change is often at variance with the actual—and sobering—facts. The present study, therefore, aims to provide an initial and important impetus in this direction.

## Figures and Tables

**Table 1:** Natural attraction and combinations of health tourism services and touristic products offered per case study.

Destinations	Aix-les-Bains (FR)	Bad Gastein (AT)	Bad Ragaz (CH)	Levico Terme (IT)	Scheidegg (DE)	Topolšica (SI)
Natural attraction	water	water, air, forest	water, air, forest	water, air, forest	water, air, forest	water
Summer season: health tourism products and…	hiking, biking, gastronomy, culture, events	hiking, gastronomy, culture	hiking, biking, gastronomy	hiking	hiking, biking	hiking, biking, culture
Winter season: health tourism products and…	winter hiking, cross-country skiing, skiing, gastronomy, culture, events	winter hiking, cross-country skiing, skiing, gastronomy	winter hiking, cross-country skiing, skiing, gastronomy		winter hiking, cross-country skiing	winter hiking, skiing, culture

**Table 3:** Health tourists’ age structure per case study.

Destinations	Aix-les-Bains (FR)	Bad Gastein (AT)	Bad Ragaz (CH)	Levico Terme (IT)	Scheidegg (DE)	Topolšica (SI)
<29 years		10%	10%	5%	20%	15%
30–49 years	40%	20%	25%	15%	50%	30%
50–69 years	50%	60%	50%	60%	20%	40%
>70 years	10%	10%	15%	20%	10%	15%

**Table 4:** Health tourists’ origin per case study.

Destinations	Aix-les-Bains (FR)	Bad Gastein (AT)	Bad Ragaz (CH)	Levico Terme (IT)	Scheidegg (DE)	Topolšica (SI)
Domestic	100%	80%	60%	90%	95%	70%
Foreign		20%	40%	10%	5%	30%
*Of these:*						
Germany		20%	12%	8%		3%
Austria			1%	2%	2%	15%
Italy			3%			5%
Switzerland					3%	1%
France			2%			
Others			22%			6%

**Table 5:** Information on the destination’s website regarding health tourism.

Information Criteria	Central Statement	Health Touristic Keywords	Prominence of Health Tourism Aspects	Presence of Evidence-Based Studies
Aix-les-Bains (FR)	Aix-Les-Bains—Riviera of the Alps	“Health” on second subpage level	Low	No
Bad Gastein (AT)	Gastein—Your vacation in the state of Salzburg, Austria	“Baths” on homepage	Medium	Third subpage level
Bad Ragaz (CH)	Bad Ragaz—The spa with global format	“Spa” on homepage	High	No
Levico Terme (IT)	Wellness with elegant flair located in scenic surroundings	“Spa town” on homepage	High	No
Scheidegg (DE)	State-approved climatic health resort Premium-Class	“Climatic health resort Premium Class” on homepage	High	No
Topolšica (SI)	Young mining town with a green heart	“Thermal springs” on homepage	Medium	Reference, but no more precise details

Source: own compilation based on [59,61,63,67,68,72].

## Data Availability

Restrictions apply to the availability of the used data. Some data was obtained from the DMOs of all case studies and are available on request from them. This applies to the references [60,62,64,65,69,71]. Furthermore, publicly available datasets were analyzed in this study that do not issue DOIs. This applies to the following references [59,61,63,66,67,68,70,72].
